# Association of the functionally significant polymorphisms of the *MMP9* gene with *H*. *pylori*-positive gastric ulcer in the Caucasian population of Central Russia

**DOI:** 10.1371/journal.pone.0257060

**Published:** 2021-09-07

**Authors:** Volodymyr Dvornyk, Irina Ponomarenko, Oksana Minyaylo, Evgeny Reshetnikov, Mikhail Churnosov

**Affiliations:** 1 Department of Life Sciences, College of Science and General Studies, Alfaisal University, Riyadh, Saudi Arabia; 2 Department of Medical Biological Disciplines, Belgorod State University, Belgorod, Russia; Indiana University Purdue University at Indianapolis, UNITED STATES

## Abstract

**Background and purpose:**

The study analyzed the association of functionally significant polymorphisms of matrix metalloproteinases (*MMPs*) genes with the development of gastric ulcer (GU) in Caucasians from Central Russia.

**Methods:**

The 781 participants, including 434 patients with GU (196 *Helicobacter pylori* (*H*. *pylori*)-positive and 238 *H*. *pylori*-negative) and 347 controls (all *H*. *pylori*-negative) were recruited for the study. Ten SNPs of the *MMP1* (rs1799750), *MMP2* (rs243865), *MMP3* (rs679620), *MMP8* (rs1940475), and *MMP9* (rs3918242, rs3918249, rs3787268, rs17576, rs17577, and rs2250889) genes were considered for association with GU using multiple logistic regression. The SNPs associated with GU and loci linked (r^2^≥0.8) to them were analyzed *in silico* for their functional assignments.

**Results:**

The SNPs of the *MMP9* gene were associated with *H*. *pylori*-positive GU: alleles C of rs3918249 (OR = 2.02, p_perm_ = 0.008) and A of rs3787268 (OR = 1.60–1.82, p_perm_ ≤ 0.016), and eight haplotypes of all studied *MMP9* gene SNPs (OR = 1.85–2.04, p_perm_ ≤ 0.016) increased risk for *H*. *pylori*-positive GU. None of the analyzed SNPs was independently associated with GU and *H*. *pylori*-negative GU. Two haplotypes of the *MMP9* gene (contributed by rs3918242, rs3918249, rs17576, and rs3787268) increased risk for GU (OR = 1.62–1.65, p_perm_ ≤ 0.006). Six loci of the *MMP9* gene, which are associated with *H*. *pylori*-positive GU, and 65 SNPs linked to them manifest significant epigenetic effects, have pronounced eQTL (17 genes) and sQTL (6 genes) values.

**Conclusion:**

SNPs of the *MMP9* were associated with *H*. *pylori*-positive GU but not with *H*. *pylori*-negative GU in Caucasians of Central Russia.

## Introduction

Gastric ulcer (GU), a disease occurring in the stomach mucosa, is the mucosal inflammation and necrotic lesion that extend to the underlying smooth muscles and are caused by multiple pathogenic factors [1]. GU is a common disease affecting about 10% of population [2]. Common causes of GU include *Helicobacter pylori* (*H*. *pylori*) infection (70–80% of GU patients), intake of non-steroidal anti-inflammatory drugs (NSAIDs), and the digestion of the gastric mucous by gastric acid/pepsin [3, 4].

The development of GU is a complex process that involves secretion of acids, generation of the reactive oxygen species, inhibition of prostaglandins, and degradation of the extracellular matrix (ECM) [5]. Damage of gastric mucosa is associated with ECM degradation in which matrix metalloproteinases (MMPs) play a key role [6]. MMPs are calcium-dependent endopeptidases involved in various processes, including ECM remodeling, cell proliferation, and inflammation. MMPs are synthesized and secreted by gastric epithelial cells, neutrophils, and macrophages [7]. Remodeling of the ECM by MMPs is thought to be one of the important factors contributing to gastric ulceration [8, 9]. Several animal studies were focused on the role of MMPs in GU [9–11]. There is evidence that MMP9 is important in the early stage of chronic GU [12].

Genetic variation in the *MMP* genes may be an important element of a complex genetic risk profile that determines the development of GU in chronic *H*. *pylori* infection [13]. *H*. *pylori* infection can increase the MMP3, MMP7, and MMP9 levels in the gastric mucosa and sera [14–16]. The significantly higher levels of the MMP9 protein were observed in *H*. *pylori*-positive GU than in the *H*. *pylori*-negative one [17].

Despite the solid evidence for the important role of MMP in GU, an association of MMP polymorphisms with GU has been studied poorly: there are only very few studies on this problem [13, 18]. The lack of experimental evidence about the possible association of the *MMPs* with GU prompts for filling this gap.

This study analyzed polymorphisms of the *MMP1*, *MMP2*, *MMP3*, *MMP8*, and *MMP9* genes for the association and possible role in the development of GU in a Caucasian sample from Central Russia.

## Materials and methods

### Study subjects

Given the available data about the allele frequencies of the *MMP* gene polymorphisms in patients with GU and controls [13], we calculated that sample size of 700 should be sufficient to ensure the statistical power of 0.80 at α = 0.05 significance level. In total, 781 participants, including 434 patients with GU and 347 controls, were recruited for the study. The participants were enrolled according to the inclusion criteria: birthplace in Central Russia and Russian ethnicity (self-reported) [19, 20].

All participants were examined by qualified gastroenterologists. GU and complications (if any) were diagnosed by conventional clinical and endoscopic examinations. The control group consisted of healthy individuals without any symptoms of gastrointestinal disease [21]. Endoscopy was not performed in healthy individuals because of both ethical reasons and the low probability of finding an active ulcer in patients without the symptoms [22]. Patients and control group volunteers having used NSAIDs, corticosteroids, and aspirin for a long-term treatment were excluded.

The *H*. *pylori* infection in patients was diagnosed by positive findings on histologic examination of biopsies obtained during endoscopy procedures by a certified pathologist and using the Giemsa stain protocol [23]. Among 434 patients with GU, 196 were *H*. *pylori*-positive and 238 were *H*. *pylori*-negative. In controls, the presence of *H*. *pylori* was determined using a commercial IgG ELISA kit (Plate Helicobacter IgG, Roche). Control group volunteers with the presence of *H*. *pylori* infection were excluded.

The study protocol was approved by the Medical Institution Ethics Committee of Belgorod State University. All study participants signed informed consent prior to enrolment in the study. The clinical and endoscopic examination of the participants was conducted at the Gastroenterology Division of Belgorod Regional Clinical Hospital.

### DNA isolation and genotyping assay

Whole blood samples (5 mL) were collected from all study participants into EDTA‐containing tubes and maintained at − 20°C until processed [24, 25]. Genomic DNA was extracted from the buffy coat by the phenol/chloroform method as described earlier [26].

Ten SNPs of the *MMP* genes (rs1799750 *MMP1*, rs243865 *MMP2*, rs679620 *MMP3*, rs1940475 *MMP8*, rs3918242, rs3918249, rs3787268, rs17576, rs17577, and rs2250889 *MMP9*) were selected for this study according to the criteria [27, 28]: previously reported associations with digestive diseases (gastric and duodenal ulcer, gastric cancer, etc.), regulatory potential, and MAF > 0.05.

All selected SNPs had significant regulatory potential as evidenced by the HaploReg online tools [29] (S1 Table); eight polymorphisms were associated with digestive diseases (gastric and duodenal ulcer, gastric and esophageal cancer, digestive cancers, gastritis) (including two SNPs associated with the peptic ulcer) in previously published candidate gene association studies (S2 Table). Two SNPs (rs3918249 and rs3787268 *MMP9*) did not demonstrate a significant association with digestive diseases but had significant regulatory potential (according to HaploReg).

The genotyping was performed using the MassARRAY® 4 System by Agena Bioscience®. Blind replicates were genotyped to control the quality [30]. Laboratory personnel that conducted genotyping was blinded to patients’ information. The repeatability test was performed for 5% of randomly selected samples, yielded 100% reproducibility.

### Statistical and functional analysis

The observed allele and genotype frequencies were checked for the correspondence to the Hardy-Weinberg equilibrium using the chi-square test [31]. Associations of the SNPs with GU were analyzed using the logistic regression and assuming dominant, log-additive, and recessive genetic models [32]. The regression analysis was adjusted for covariates: BMI as a quantitative variable, whereas a family history of peptic ulcer, alcohol and tobacco consumption, stress, the presence of cardiovascular and endocrine pathology were applied as qualitative parameters (Table 1). The given sample size (434 patients with GU and 347 controls) was sufficient to detect differences in allelic frequencies between the affected subjects and controls at OR = 1.33–1.82 for the additive model, OR = 1.58–1.86 for the dominant model and OR = 1.61–27.0 for the recessive model (at 80% power, ɑ = 0.05 for 2-sided test). Statistical power for each SNP was estimated using Quanto 1.2.4 [33]. The haplotype blocks were identified using the «Solid Spine» algorithm (D’ > 0.8) as implemented in HaploView v.4.2 [34]. The association analyses and adjustment for multiple comparisons by the adaptive permutation test [35] were conducted using the PLINK v. 2.0 software [36]. The significance value was set at p_perm_<0.017 (after the Bonferroni correction based on the numbers of paired comparisons, n = 3: GU–control, *H*. *pylori*-positive GU—control, and *H*. *pylori*-negative GU—control).

**Table 1:** Phenotypic characteristics of the study participants.

Parameters	Control	GU	p
	mean ± SD, % (n)	mean ± SD, % (n)	
N	347	434	-
Age, years (min–max)	48.47±13.69 (22–79)	49.08±11.18 (22–79)	0.36
Gender ratio, f/m	66.28/33.72 (230/117)	68.66/31.34 (298/136)	0.53
BMI, kg/m2	26.83±5.09	27.93±5.02	**0.003**
Age of developing peptic ulcer, years	-	45.47±12.08	-
Family history of peptic ulcer	4.32 (15)	17.05 (74)	**0.0005**
Current smoking	14.99 (52)	25.35 (110)	**0.001**
Alcohol consumption	32.28 (112)	49.31 (214)	**0.0005**
Stress	37.17 (129)	79.26 (344)	**0.0005**
Positivity *H*. *pylori* test (endoscopic biopsy and histological identification)	-	45.16 (196)	-
Anatomical characteristics of the ulcer			
Location			
Stomach: Body	-	5.07 (22)	-
Pylorus	-	5.53 (24)	-
Antrum	-	89.40 (388)	-
Sizes ulcer (diameter) (cm)	-	0.50±0.36	-
Sizes ulcer: Small (<0.5 cm)	-	64.06 (278)	-
Medium (0.5–1.0 cm)	-	28.11 (124)	-
Large (>1.0 cm)	-	7.83 (32)	-
Associated complications			
Bleeding	-	1.38 (6)	-
Perforation	-	5.99 (26)	-
Stenosis	-	2.30 (10)	-
Malignancy	-	3.23 (14)	-
Somatic pathologies			
Cardiovascular pathology	26.80 (93)	60.83 (264)	**0.0005**
Endocrine pathology	3.17 (11)	7.37 (32)	**0.02**
Kidney pathology	2.59 (9)	4.61 (20)	0.20
Respiratory system pathology	4.32 (15)	5.53 (24)	0.55
Nervous system pathology	7.78 (27)	9.68 (42)	0.42
Musculoskeletal system pathology	6.91 (24)	8.29 (36)	0.56

P values <0.05 are shown in bold.

The functional importance (missense replacement, eQTLs and sQTLs, regulatory potential) of the genetic variants associated with GU and those linked to them was studied *in silico* [37, 38]. The SIFT online tool [39] was used to identify missense replacements and predict their functional effects. The SNP epigenetic effects were analyzed using RegulomeDB [40] and HaploReg [29]. The data from the GTExportal browser [41] was used to estimate the influence of the GU-candidate loci on mRNA levels and splicing QTLs. Likewise, regulatory potential, eQTL and sQTL values of polymorphisms in strong linkage disequilibrium (LD, r^2^≥0.8) with the GU-associated loci were estimated [42, 43]. The linked SNPs were identified using HaploReg [29].

## Results

Baseline and clinical characteristics of the participants are presented in Table 1. The GU patients had higher BMI (p = 0.003), higher percentage of positive family history of peptic ulcer (p = 0.0005), alcohol (p = 0.0005) and tobacco (p = 0.001) consumption, stress (p = 0.0005), the presence of cardiovascular (p = 0.0005) and endocrine (p = 0.02) pathology as compared to the healthy participants (the data are shown in Table 1). Therefore, these parameters were used as confounding factors in the logistic regression analyses.

**Fig 1:**
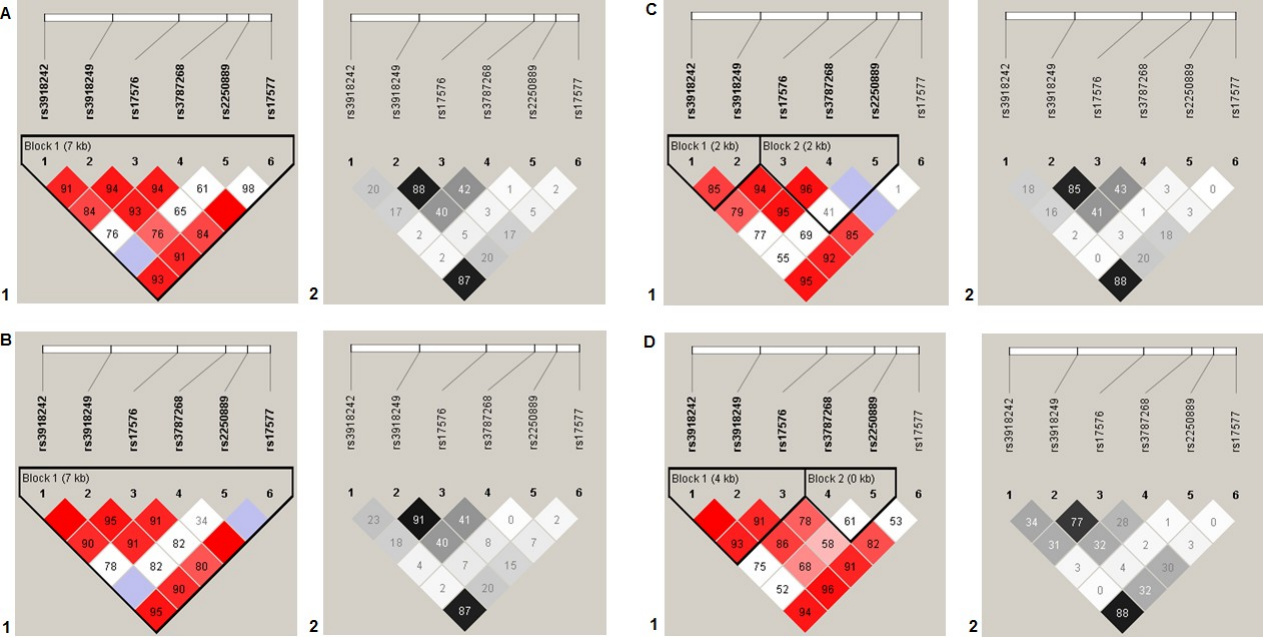
Linkage disequilibrium (LD) between SNPs rs3918242, rs3918249, rs17576, rs3787268, rs2250889 and rs17577 of the *MMP9* gene in GU patients. A, summary; B, *H*. *pylori*-positive GU patients; C, *H*. *pylori*-negative GU patients; D, control group. LD values are given as Lewontin’s standardized coefficient D′ (Figure sections 1) and the square of the Pearson’s correlation coefficient (r^2^) (Figure sections 2) between SNPs.

The summary data about the studied SNPs is given in S3 Table. All SNPs corresponded to the HWE (p>0.005, p_bonf_>0.05). None of the SNPs was independently associated with GU according to any of the genetic models (Table 2). As to *H*. *pylori*-positive GU, only loci of the *MMP9* gene manifested association with the disease. Allele C at rs3918249 *MMP9* locus was associated with *H*. *pylori*-positive GU according to the dominant model (OR = 2.02, 95% CI 1.21–3.37, p_perm_ = 0.008, power—94.68%), and allele A at rs3787268 *MMP9* was associated with *H*. *pylori*-positive GU according to the additive (OR = 1.60 95% CI 1.09–2.34, p_perm_ = 0.016, power = 89.98%) and dominant (OR = 1.82 95% CI 1.14–2.89, p_perm_ = 0.012, power = 91.14%) genetic models (the data are provided in Table 3). Several haplotypes of the studied SNPs *MMP9* gene were associated with GU (four SNPs within two haplotypes, OR = 1.62–1.65, p_perm_ ≤ 0.006) and *H*. *pylori*-positive GU (all six SNPs within eight haplotypes, OR = 1.85–2.04, p_perm_ ≤ 0.016) (Table 4, Fig 1). None of the SNPs was associated with *H*. *pylori*-negative GU either independently or within haplotypes (Table 3).

**Table 2:** Associations of the *MMP* gene polymorphisms with GU.

SNP	Gene	MAF	n	Additive model				Dominant model					Recessive model			
				OR	95%CI		Р	OR		95%CI		Р	OR	95%CI		Р
					L95	U95				L95	U95			L95	U95	
rs1940475	*MMP8*	T	776	0.96	0.76	1.22	0.733	0.89	0.61		1.31	0.569	1.00	0.67	1.50	0.989
rs1799750	*MMP1*	2G	751	0.87	0.68	1.11	0.257	0.80	0.55		1.18	0.261	0.85	0.56	1.30	0.457
rs679620	*MMP3*	T	773	1.01	0.79	1.30	0.917	1.06	0.71		1.58	0.793	0.98	0.65	1.47	0.924
rs243865	*MMP2*	T	763	0.99	0.75	1.31	0.931	0.95	0.67		1.36	0.789	1.11	0.57	2.17	0.765
rs3918242	*MMP9*	T	767	0.76	0.54	1.08	0.131	0.76	0.51		1.12	0.165	0.56	0.17	1.82	0.336
rs3918249	*MMP9*	C	767	1.12	0.87	1.44	0.367	1.45	1.00		2.09	0.049	0.79	0.48	1.30	0.363
rs17576	*MMP9*	G	776	1.17	0.91	1.51	0.225	1.27	0.89		1.82	0.194	1.16	0.71	1.90	0.564
rs3787268	*MMP9*	A	775	1.23	0.90	1.67	0.188	1.37	0.96		1.96	0.081	0.76	0.29	1.95	0.564
rs2250889	*MMP9*	G	770	0.89	0.62	1.29	0.533	0.90	0.59		1.37	0.616	0.69	0.20	2.31	0.546
rs17577	*MMP9*	A	760	0.76	0.54	1.07	0.120	0.79	0.54		1.18	0.252	0.33	0.09	1.19	0.090

All results were obtained after adjustment for covariates. ОR, odds ratio; 95%CI, 95% confidence interval.

**Table 3:** Associations of the *MMP* gene polymorphisms with *H*. *pylori*-positive and *H*. *pylori*-negative GU.

SNP	Gene	MAF	n	Additive model				Dominant model					Recessive model			
				OR	95%CI		Р	OR		95%CI		Р	OR	95%CI		Р
					L95	U95				L95	U95			L95	U95	
*H*. *pylori*-positive GU																
rs1940475	*MMP8*	T	540	0.97	0.71	1.32	0.833	0.86	0.52		1.42	0.556	1.07	0.63	1.81	0.796
rs1799750	*MMP1*	2G	523	0.81	0.59	1.12	0.212	0.79	0.48		1.30	0.345	0.71	0.40	1.28	0.254
rs679620	*MMP3*	T	537	0.91	0.65	1.26	0.568	0.96	0.57		1.63	0.887	0.80	0.46	1.39	0.426
rs243865	*MMP2*	T	533	0.94	0.64	1.37	0.742	0.87	0.54		1.40	0.563	1.15	0.47	2.81	0.752
rs3918242	*MMP9*	T	533	0.87	0.56	1.36	0.539	0.94	0.57		1.56	0.811	0.28	0.03	2.23	0.228
rs3918249	*MMP9*	C	533	1.30	0.94	1.80	0.115	**2.02**	**1.21**		**3.37**	**0.007**	0.84	0.44	1.60	0.600
rs17576	*MMP9*	G	538	1.45	1.04	2.02	0.028	1.76	1.07		2.89	0.026	1.47	0.80	2.71	0.215
rs3787268	*MMP9*	A	539	**1.60**	**1.09**	**2.34**	**0.016**	**1.82**	**1.14**		**2.89**	**0.012**	1.52	0.55	4.17	0.419
rs2250889	*MMP9*	G	532	0.97	0.60	1.57	0.910	0.99	0.57		1.73	0.984	0.79	0.16	3.79	0.766
rs17577	*MMP9*	A	526	0.91	0.59	1.42	0.684	1.03	0.62		1.70	0.921	0.24	0.03	1.89	0.175
*H*. *pylori*-negative GU																
rs1940475	*MMP8*	T	582	0.97	0.72	1.30	0.825	0.96	0.60		1.54	0.858	0.95	0.58	1.56	0.852
rs1799750	*MMP1*	2G	567	0.92	0.69	1.23	0.573	0.82	0.52		1.31	0.414	0.98	0.59	1.62	0.923
rs679620	*MMP3*	T	581	1.09	0.81	1.48	0.559	1.13	0.69		1.85	0.630	1.13	0.69	1.83	0.635
rs243865	*MMP2*	T	573	1.03	0.73	1.45	0.856	1.02	0.66		1.58	0.923	1.11	0.49	2.51	0.799
rs3918242	*MMP9*	T	577	0.68	0.44	1.06	0.089	0.62	0.37		1.03	0.064	0.80	0.21	2.99	0.740
rs3918249	*MMP9*	C	579	0.97	0.71	1.31	0.838	1.01	0.71		1.71	0.671	0.73	0.39	1.37	0.331
rs17576	*MMP9*	G	584	0.97	0.71	1.32	0.839	0.98	0.64		1.51	0.933	0.91	0.48	1.72	0.775
rs3787268	*MMP9*	A	581	0.93	0.63	1.37	0.708	1.05	0.68		1.63	0.816	0.18	0.02	1.43	0.106
rs2250889	*MMP9*	G	580	0.83	0.52	1.31	0.421	0.82	0.49		1.39	0.469	0.63	0.13	3.03	0.566
rs17577	*MMP9*	A	574	0.64	0.41	1.00	0.050	0.62	0.37		1.03	0.067	0.39	0.09	1.80	0.228

All results were obtained after adjustment for covariates. ОR, odds ratio; 95%CI, 95% confidence interval.

**Table 4:** Significant associations of the *MMP9* gene haplotypes with GU and *H*. *pylori*-positive GU.

SNPs	Haplotype	Frequency		OR	P_raw value_	P_perm_
		Cases	Controls			
GU						
rs3918242-rs3918249-rs17576	CCG	0.2568	0.1846	1.65	0.002	0.004
rs3918242-rs3918249-rs17576-rs3787268	CCGA	0.2306	0.1679	1.62	0.004	0.006
*H*. *pylori*-positive GU						
rs3918242-rs3918249-rs17576	CCG	0.2827	0.1841	1.92	0.001	0.005
rs3918242-rs3918249-rs17576-rs3787268	CCGA	0.2650	0.1639	2.04	0.0007	0.002
rs3918242-rs3918249-rs17576-rs3787268-rs2250889	CCGAC	0.2563	0.1581	2.04	0.001	0.004
rs3918242-rs3918249-rs17576-rs3787268-rs2250889-rs17577	CCGACG	0.2511	0.1652	1.90	0.002	0.016
rs3918249-rs17576-rs3787268	CGA	0.2654	0.1721	1.88	0.003	0.011
rs3918249-rs17576-rs3787268-rs2250889-rs17577	CGACG	0.2523	0.1634	1.96	0.002	0.011
rs17576-rs3787268	GA	0.2733	0.1802	1.85	0.003	0.010
rs17576-rs3787268-rs2250889-rs17577	GACG	0.2538	0.1607	2.00	0.001	0.010

*Note*: All results were obtained after adjustment for covariates; OR, odds ratio; P, significance level.

### Functional SNP predictions

#### Non-synonymous SNPs

Among the six loci of the *MMP9* gene associated with *H*. *pylori*-positive GU, three SNPs were missense variant: rs17576 (amino acid change Gln279Arg, SIFT score 0.29, SIFT prediction «tolerated»), rs2250889 (Arg574Pro, SIFT score 1.00, SIFT prediction «tolerated») and rs17577 (Arg668Gln, SIFT score 0.02, SIFT prediction «deleterious»).

#### Regulatory effects predictions

According to RegulomeDB and HaploReg, all six SNPs of the *MMP9* gene (rs3918242, rs3918249, rs17576, rs3787268, rs2250889 and rs17577) associated with *H*. *pylori*-positive GU possess significant regulatory effects. The RegulomeDB suggests the most significant regulatory potential for rs17577 (rank - 2b, score—0.79). The other five *H*. *pylori*-positive GU-associated SNPs (rs3918242, rs3918249, rs17576, rs3787268, rs2250889) have RegulomeDB rank = 4–5 and RegulomeDB score = 0.59–0.61. According to the HaploReg database, the above six polymorphisms were located in DNase I hypersensitive sites, five SNPs–in the specific region of DNA binding with modified histone marking promoters (rs3918249, rs17576, rs3787268, rs2250889 and rs17577) and enhancers (rs3918242, rs3918249, rs17576, rs3787268 and rs17577) in various tissues, and in the sixteen motifs to the transcription factors (TFs), three SNPs (rs17576, rs2250889 and rs17577)—in evolutionarily conserved DNA segments, and two SNPs (rs2250889 and rs17577)—in the protein-bound site (S1 Table). Herewith, alleles of the *MMP9* gene loci associated with increased risk for *H*. *pylori*-positive GU (Table 4) increases affinity to twelve TFs (Ahr:Arnt, HIF1, Myc, Hmx, Hoxb8, HDAC2, Mef2, Pou1f1, Sox, Zfp105, p300, NRSF) and decreases affinity to the four TFs (E2F, Arid3a, Pax-5, Pax-4) (S4 Table).

In addition to the six *H*. *pylori*-positive GU-related SNPs, regulatory significance was estimated for 65 loci linked to them (the data are provided in S5 Table). Seventeen SNPs have been positioned in evolutionarily conserved DNA regions. Nine SNPs (including four synonymous and five missense replacements) were located in protein-coding regions (exons) of the *MMP9* gene, 26 in introns, and 30 in intergenic areas. All 65 SNPs linked to the *H*. *pylori*-positive GU-associated SNPs had a significant regulatory potential; several polymorphisms manifested epigenetic effects (S5 Table). For example, rs6073989 (linked to rs3787268, r^2^ = 0.95) is positioned in the specific area of DNA binding with modified histone marking promoters (H3K4me3, H3K9ac) and enhancers (H3K4me1, H3K27ac) in more than ten tissues/organs, the DNAase I hypersensitive segments in sixteen tissues/organs, and a sites of five regulatory DNA motifs (CAC-binding-protein, EWSR1-FLI1, PRDM1, SP1, TATA). The SNP rs6073991, which also was in linkage disequilibrium with rs3787268 (r^2^ = 0.95), was located in the hypersensitive region to DNAase-I in 49 (!) tissues/organs, in the protein-bound region (with this DNA region interact three regulatory proteins—NRSF, ZNF143, BCL3), and a putative transcription factor binding sites (SZF1-1, T3R) (S5 Table). Importantly, the epigenetic effects of the *H*. *pylori*-positive GU-associated SNPs and 65 polymorphisms linked to them of the *MMP9* gene were reported for the target organs of GU, adult stomach mucosa and smooth muscle, fetal stomach.

#### Expression quantitative trait loci (eQTLs)

Referring to the data of the GTExportal resource, six *H*. *pylori*-positive GU-associated polymorphisms *MMP9* gene and 61 SNPs linked to them had the eQTL significance (*cis*-eQTL and *trans*-eQTL) and correlated with mRNA levels of 17 genes in more than 25 various tissues and organs (*NEURL2*, *SLC12A5*, *CD40*, *MMP9*, *RP3-337O18*.*9*, *NTTIP1*, *PCIF1*, *SPATA25*, *RP11-465L10*.*10*, *ZSWIM1*, *RPL13P2*, *SNX21*, *WFDC10B*, *PLTP*, *SYS1*, *WFDC3*, *ZNF335*) (S6 and S7 Tables). For example, rs3787268 and rs3918249 (individually associated with *H*. *pylori*-positive GU) may affect the expression genes (*RP3-337O18*.*9*, *PLTP*, *NEURL2*) in the organs of the digestive system (esophagus, various sections of the colon) as well as other tissues/organs related to the development of GU: brain (frontal cortex and pituitary (*PLTP*)), adipose tissue (visceral and subcutaneous) (*NEURL2*, *SPATA25*, *PLTP*, *SLC12A5*, *CD40*, *ZSWIM1*, *RP3-337O18*.*9)*, whole blood (*ZNF335*), thyroid (*PLTP*, *NEURL2*), adrenal gland (*PLTP*, *PCIF1*, *SLC12A5*, *RP11-465L10*.*10)*, etc. Importantly, the risk alleles of the analyzed loci (e.g., A rs3787268 and C rs3918249 C) are usually associated with the lower expression of the genes (S6 Table).

#### Splicing quantitative trait loci (sQTLs)

Analysis of the GTExportal data suggested that the six GU-associated loci of *MMP9* had significant sQTL values and might influence alternative splicing of five genes (*SLC12A5*, *ACOT8*, *CD40*, *SNX21*, *PLTP*) in various tissues and organs (the results are shown in S8 Table). These polymorphisms manifested strong linkage to 61 SNPs, which affect splicing QTL of six genes (*CD40*, *SLC12A5*, *ACOT8*, *SNX21*, *PLTP*, *SLC35C2*) in more than 15 organs and tissues (S9 Table). Importantly, the independently associated with *H*. *pylori*-positive GU loci rs3787268 and rs3918249 correlate with splicing QTLs of the *PLTP* gene in subcutaneous adipose tissue and *SLC12A5* gene in the various parts of the brain (e.g., cortex and substantia nigra, pituitary), which are known to play a role in the pathogenesis of GU. Interestingly, the GU risk allele, C rs3918249, correlates with the elevated level of in the sQTL of the *SLC12A5* gene (β>0) in the substantia nigra and pituitary and with the lower level of the sQTL of the *SLC12A5* gene (β<0) in the brain cortex. The GU risk allele A rs3787268 is associated with the higher level of the sQTL of the *PLTP* gene in the subcutaneous adipose tissue (S8 Table). Besides, rs3787268 and rs3918249 each are in strong LD with eleven sQTL SNPs (S9 Table).

## Discussion

In the present study, we report for the first time the association of the SNPs rs3918249 and rs3787268 of *MMP9* with *H*. *pylori*-positive GU but not with *H*. *pylori*-negative GU in the Caucasian population of Central Russia. Alleles C of rs3918249 *MMP9* (OR = 2.02) and A of rs3787268 *MMP9* (OR = 1.60–1.82), and eight haplotypes of the six studied SNPs of the *MMP9* gene (OR = 1.85–2.04) increased risk for *H*. *pylori*-positive GU. None of the *MMP* gene SNPs was independently associated with GU. Also, two haplotypes of the *MMP9* gene (contributed by four SNPs, rs3918242, rs3918249, rs17576, and rs3787268) increased risk for GU (OR = 1.62–1.65). Six loci of the *MMP9* gene, which are associated with *H*. *pylori*-positive GU, and 65 SNPs linked to them manifest significant epigenetic effects, have pronounced eQTL (17 genes) and sQTL (six genes) values in the organs of the digestive system and the other tissues/organs, which have been suggested to contribute to GU.

Only two genome-wide association studies (GWAS) of the peptic ulcer disease (PUD) have been conducted so far [44, 45]. One of them reported only two SNPs (rs2294008 *PSCA* and rs505922 *ABO*) associated with duodenal ulcer in Japanese [44]; another determined eight PUD-associated loci in the *MUC1*, *MUC6*, *FUT2*, *PSCA*, *ABO*, *CDX2*, *GAST* and *CCKBR* genes, including the two reported for the Japanese cohort [45]. While the estimated heritability for PUD is about 28% [45], only 6% of the estimated variance in the trait is attributed to genome-wide common SNPs (i.e., SNP-based heritability) [45] that raises a problem of so-called “missing heritability”. This problem may be addressed by studying associations of candidate genes for peptic ulcer. In these terms, the *MMPs* genes are very good candidates, as they are related to both the pathogenesis of *H*. *pylori-*associated gastric ulcer and the inflammatory response of the mucosa [13].

So far, only one study [13] reported a significant association of an *MMP9* gene variant with *H*. *pylori*-positive GU. The authors analyzed 20 SNPs of the *MMP1*, *3*, *7*, and *9* genes in the sample of 599 *H*. *pylori*-infected German patients and determined that two SNPs were associated with the disease: the rs17576 polymorphism of the *MMP9* gene and another variant in the promoter region of the *MMP7* gene. Interestingly, Hellmig S. et al. determined allele A of rs17576 as a risk factor for the disease [13], while our results suggested allele variant G of the same locus (within the significantly associated haplotypes of the *MMP9* gene) as the risk factor for the Caucasian population of Central Russia. Yeh et al. [18] did not find any significant association of the above SNP with the disease in a Taiwanese population. Thus, our study is the first that reports the association of rs3918242, rs3918249, rs3787268, rs2250889, rs17577 of the *MMP9* gene with *H*. *pylori*-positive GU.

There are quite a few studies, which analyzed the association of *MMP* gene polymorphisms with gastric cancer (S2 Table) and reported such an association for the *MMP9* gene SNPs included in our study (rs3918242, rs17576, rs17577). Notably, allele C of rs3918242, a risk factor for *H*. *pylori*-positive GU in Caucasians from Central Russia in the present study, also increased the risk for gastric cancer [46, 47] and esophageal cancer [48]. It is thought that *H*. *pylori*-associated GU is positively linked to stomach cancer due to the damage of gastric mucosa [49] and the *MMP9* gene may be one of the candidate genes involved in both *H*. *pylori*-associated GU and gastric cancer [50].

The *MMP9* gene is located on chromosome 20q11.1–13.1. The encoded protein (type IV collagenase, also known as gelatinase B) can degrade various collagen (collagen types IV, V, VII, X, XIV, gelatin) and non-collagen substrates (elastin, aggrecan, fibronectin, nidogen, laminin, etc.) of the extracellular matrix (ECM) [51]. ECM of the gastric mucosa contains a significant proportion of collagen, elastin, fibronectin, laminin, hyaluronic acid, and proteoglycan, and their degradation by MMPs is important for the stability of the cellular microenvironment [13]. MMP9 is a key enzyme implicated in gastric ulcer [52, 53]. ECM degradation by MMPs is apparently a key factor contributing to gastric mucosal damage [6]. The significant elevation of the MMP9 level in gastric ulcer tissues suggested that the enzyme may regulate mucosa lesions in GU by degrading collagens and creating lesions [50]. Also, this enzyme is important in the early phase of chronic GU [12].

The present study reports association of the *MMP9* gene polymorphisms with *H*. *pylori*-positive GU but not with *H*. *pylori*-negative GU. The available literature also suggests that *MMP9* gene polymorphisms apparently contribute to a genetic risk profile to develop GU in chronic *H*. *pylori* infection [13]. There is evidence that MMPs can be induced by both *H*. *pylori* bacterial products and proinflammatory cytokines [54]. MMPs have elevated expression in gastric epithelial cells infected with *H*. *pylori* that might contribute to the GU pathogenesis. Li et al. [17] analyzed samples of gastric mucosa from the antrum and ulcer site and found that the higher MMP9 expression was associated with the *H*. *pylori* infection and correlated with the level of inflammation determined histologically at the border of the ulcer. Several studies demonstrated a correlation between the elevated serum levels of MMP9 and higher MMP9 activity in antral mucosa of *H*. *pylori*-infected patients with gastritis [55, 56]. Antral mucosa of *H*. *pylori*-infected subjects demonstrates a 19-fold higher MMP9 protein activity than that of uninfected individuals [55] as *H*. *pylori* induces NF-kappaB activation through the intracellular signaling pathway resulting in transcription of the *MMP*9 gene [15]. Notably, the *H*. *pylori*-induced *MMP9* up-regulation can be reversed after successful pathogen eradication. If the eradication failed, no difference in the MMP9 protein expression was detected in epithelial cells and fibroblasts before and after the treatment [57]. *H*. *pylori* plays an important role not only in gastroduodenal diseases (chronic gastritis, peptic ulcer, gastric cancer, etc.) but also in various extragastric pathologies (cardiovascular, endocrine (insulin resistance, diabetes mellitus), etc.) [58]. Indeed, higher BMI, the higher prevalence of cardiovascular and endocrine pathologies, among GU patients (including 45.16% of *H*. *pylori*-positive) as compared to the controls (*H*. *pylori*-negative) was documented in the present study (Table 1). Importantly, polymorphisms of the *MMP* genes (including *MMP9*) were suggested to determine the susceptibility to cardiovascular diseases and their complications in various populations [59–61], including Caucasians of Central Russia [42, 62, 63]. Wu et al. [45] documented significant positive SNP-based genetic correlation (r_g_) between PUD and BMI, body fat-related traits, and coronary artery disease. The *in silico* analysis in our study also identified significant expression and splicing QTLs among the *H*. *pylori*-positive GU-associated SNPs of the *MMP9* gene and their proxies affecting several genes (e.g., *PLTP*, *CD40*, *SLC12A5*, *NEURL2*, *SPATA25*, *ZSWIM1*, *RP3-337O18*.*9*) in the adipose tissue (visceral and subcutaneous).

The available literature data suggests that the above genes are involved in biological pathways contributing to pathophysiology of GU. For example, the *PLTP* gene encodes one of two phospholipid transfer proteins found in human blood plasma. This protein transfers phospholipids and cholesterol between different classes of lipoproteins and was implicated in many disorders, including hyperlipidemia, obesity, metabolic syndrome, type II diabetes, and others [64]. There is evidence that PLTP may play important roles in the nervous system through participation in signal transduction pathways [65], control of vitamin E content [66], and maintenance of blood-brain barrier integrity [67]. Another example is the *CD40* gene. It is a member of the tumor necrosis factor receptor superfamily and encodes a protein playing a key role in a broad range of immune and inflammatory responses including T cell activation, immunoglobulin isotype switching and cytokine production [68]. *CD40* has been implicated in various disorders, such as atherosclerosis, cardiovascular, metabolic (arterial hypertension, diabetes mellitus, etc.), and the others [68, 69]. Importantly, the *CD40* DNA methylation levels [70] and CD40 expression (protein and mRNA) [71] were associated with gastric cancer. The *ACOT8* gene encodes a peroxisomal thioesterase (acyl-CoA thioesterase 8) involved in the oxidation of fatty acids [72]. This enzyme is localized ubiquitously throughout all cellular compartments and is among the key enzymes of lipid metabolism [72].

Due to their pleiotropism, polymorphisms of the *MMP9* genes play a key role in multiple biological pathways and therefore have been implicated not only in peptic ulcer [13, 73, present study], but also in cardiovascular diseases [60, 61] as well as a broad range of other disorders involving processes of synthesis and degradation of extracellular matrix: various cancers, including digestive [46–48, 74–76], glaucoma [27, 77, 78], and others [79–82].

One limitation of this study should be acknowledged though. Like other association studies, our study did not utilize any experimental procedures to test the predictions about the possible functional significance of the *H*. *pylori*-positive GU-associated SNPs of the *MMP9* gene, because “wet” experiments were beyond the study scope. The predictions were made based solely on the *in silico* analysis of the available functional genomics databases (HaploReg [29], GTExportal browser [41], SIFT online tool [39], RegulomeDB [40]), which include data of large-scale studies in this area (Genotype-Tissue Expression (GTEx) project, Roadmap Epigenomics and ENCODE projects, etc.).

## Conclusions

The *MMP9* gene polymorphisms were associated with *H*. *pylori*-positive GU but not with *H*. *pylori*-negative GU in the Caucasian population of Central Russia. Alleles C rs3918249 *MMP9* (OR = 2.02) and A rs3787268 *MMP9* (OR = 1.60–1.82), and eight haplotypes of the all studied six SNPs *MMP9* gene (OR = 1.85–2.04) increased risk for *H*. *pylori*-positive GU. None of the *MMPs* genes SNPs was independently associated with GU. Also, two haplotypes *MMP9* gene (including the following SNPs, rs3918242, rs3918249, rs17576, and rs3787268) increased risk for GU (OR = 1.62–1.65). Six loci of the *MMP9* gene associated with *H*. *pylori*-positive GU and 65 SNPs linked to them manifest significant epigenetic effects, have noticeable eQTL (17 genes) and sQTL (6 genes) values.

## Supporting information

S1 Table The regulatory potential of the studied SNPs. (XLS)

S2 Table The literature data about associations of the studied polymorphisms of the ММР genes with some digestive diseases. (DOC)

S3 Table The allele and genotype frequencies of the studied MMP gene SNPs in the GU patients and control group. (DOC)

S4 Table Effect of the MMP-9 gene polymorphisms on affinity of the DNA regulatory motifs. (DOCX)

S5 Table Regulatory effects of the six *H*. *pylori*-positive GU-associated SNPs of the MMP9 gene and SNPs in high LD (r2≥0.80). (XLS)

S6 Table eQTL values of the *H*. *pylori*-positive GU-associated SNPs MMP-9 gene. (XLS)

S7 Table Effect of SNPs in high LD (r2≥0.80) with the *H*. *pylori*-positive GU-associated polymorphisms MMP-9 gene on gene expression level. (XLS)

S8 Table sQTL values of the *H*. *pylori*-positive GU-associated SNPs MMP-9 gene. (XLS)

S9 Table Effect of SNPs in high LD (r2≥0.80) with the *H*. *pylori*-positive GU-associated polymorphisms MMP-9 gene on alternative splicing level. (XLS)

S1 References References to S2 Table. (DOC)
