# Supplementary material for: Association of the functionally significant polymorphisms of the MMP9 gene with H. pylori-positive gastric ulcer in the Caucasian population of Central Russia
Source: PLoS One. 2021 Sep 7;16(9):e0257060. doi: 10.1371/journal.pone.0257060 (PMC8423286; doi:10.1371/journal.pone.0257060)
Supplement: S2 Table — (DOC) [file pone.0257060.s002.doc]

S2 table

The literature data about associations of the studied polymorphisms of the *ММР* genes with some digestive diseases

(peptic ulcer, gastric and esophageal cancer, digestive cancers, gastritis)

| Chr | SNP | Gene | Number of publications in  PubMed/PubMed Central | Phenotype | Association (significance)  (associated allele) | Reference |
| --- | --- | --- | --- | --- | --- | --- |
| 11 | rs1940475 | *MMP8* | 6/18 | gastric adenocarcinoma | Higher risk for recurrence (p = 0.005) and lower overall survival *(*p = 0.001), recurrence-free survival (p = 0.005) and disease-free survival (p = 0.011), lower activities of MMP8 (p = 0.011) (TT) | Lin Y. et al., 2017 |
| 11 | rs1799750 | *MMP1* | 75/133 | peptic ulcer | OR=1.94, p=0.02 (1G/2G) | Shaimardanova E.Kh. et al., 2016 |
|  |  |  |  | gastric cancer | OR=3.34, р=0.016 (2G/2G) | Devulapalli K. et al., 2014 |
|  |  |  |  | gastric cancer | OR =1.05, р=0.013 (2G) | Peng Q. et al., 2015 |
|  |  |  |  | digestive cancers | OR = 1.31, p < 0.00001 (2G) | Li X. et al., 2013 |
|  |  |  |  | gastric cancer | OR= 1.84-2.58, р<0.05 synergistic interaction with cigarette smoking and H. pylori infection | Yang MD et al., 2017 |
|  |  |  |  | gastric cancer | p<0.05 | Tian J. et al., 2019a |
|  |  |  |  | esophageal cancer | OR= 1.47, р<0.05 | Tian J. et al., 2019b |
|  |  |  |  | esophageal carcinoma | OR=2.04-3.65, p<0.05 (haplotype rs1799750- rs679620) | Guan X. et al., 2014 |
|  |  |  |  | esophageal adenocarcinoma | OR = 3.2, p < 0.001 (2G) | Cheung W.Y. et al., 2012 |
|  |  |  |  | esophageal adenocarcinoma | OR = 1.83, p = 0.005 (2G/2G) | Bradbury P.A. et al., 2009 |
| 11 | rs679620 | *MMP3* | 34/85 | gastric adenocarcinoma | Higher risk for recurrence (p = 0.04) and lower overall survival (p = 0.003), recurrence-free survival (p = 0.04) and disease-free survival (p = 0.046), highest activities of MMP3 (p = 0.015) (AA) | Lin Y. et al., 2017 |
|  |  |  |  | esophageal carcinoma | OR=1.93, p<0.01 | Guan X. et al., 2014 |
|  |  |  |  | esophageal carcinoma | OR=2.04-3.65, p<0.05 (haplotype rs1799750- rs679620) | Guan X. et al., 2014 |
| 16 | rs243865 | *MMP2* | 80/92 | esophageal cancer | OR= 0.67, р<0.05 | Tian J. et al., 2019b |
|  |  |  |  | digestive cancers | OR = 0.69, p = 0.0007 (CT or TT) | Li X. et al., 2013 |
|  |  |  |  | digestive cancer | OR=0.68, p=0.002 (TT+CT ) | Zhang L.Y. et al., 2011 |
|  |  |  |  | esophageal carcinoma | OR = 0.32, p = 0.02 | Zhang L. et al., 2015 |
|  |  |  |  | esophageal cancer | OR = 0.67, р<0.05 (T) | Peng Z. et al., 2010 |
| 20 | rs3918242 | *MMP9* | 114/140 | *H. pylori* infection in children with chronic gastritis | OR = 3.1, р<0.05 (CC) | Shan Q.W. et al., 2010 |
|  |  |  |  | duodenal ulcer in children | p>0.05 | Shan Q.W. et al., 2010 |
|  |  |  |  | chronic gastritis in children | p>0.05 | Shan Q.W. et al., 2010 |
|  |  |  |  | peptic ulcer | p>0.05 | Shaimardanova E.Kh. et al., 2016 |
|  |  |  |  | gastric cancer | OR=3.35, р<0.05 (haplotype CAA rs3918242-rs17576-rs17577) | Okada R. et al., 2017 |
|  |  |  |  | esophageal cancer | OR=2.71, р=0.02 (СС) | Zhang L. et al., 2015 |
|  |  |  |  | digestive cancers | OR = 1.42, p = 0.02 | Hu C. et al., 2018 |
|  |  |  |  | gastric cancer | OR = 1.66, р<0.05 (CC+CT) | Peng Z. et al., 2017 |
|  |  |  |  | invasive phenotype of gastric cancer | р<0.05 (T) | Matsumura S. et al., 2005 |
| 20 | rs17576 | *MMP9* | 81/101 | gastric ulcer in *H. pylori* infection | OR=2.40, p=0.013 (A) | Hellmig S. et al., 2006 |
|  |  |  |  | duodenal ulcer after *H. pylori* infection | p>0.05 | Yeh Y.C. et al., 2010 |
|  |  |  |  | gastric ulcer after *H. pylori* infection | p>0.05 | Yeh Y.C. et al., 2010 |
|  |  |  |  | gastritis after *H. pylori* infection | p>0.05 | Yeh Y.C. et al., 2010 |
|  |  |  |  | peptic ulcer | OR=0.49, p=0.007 (AA) | Shaimardanova E.Kh. et al., 2016 |
|  |  |  |  | peptic ulcer in *H. pylori* infection | OR=0.54, p=0.03 (AA) | Shaimardanova E.Kh. et al., 2016 |
|  |  |  |  | duodenal ulcer | OR=1.57, p=0.009 (AG) | Shaimardanova E.Kh. et al., 2016 |
|  |  |  |  | gastric cancer | OR=4.34, р<0.05 (Q) | Okada R. et al., 2017 |
|  |  |  |  | gastric cancer | OR=3.35, р<0.05 (haplotype CAA rs3918242-rs17576-rs17577) | Okada R. et al., 2017 |
| 20 | rs2250889 | *MMP9* | 22/38 | gastric cancer | p>0.05 | Kim J.H. et al., 2011 |
|  |  |  |  | esophageal carcinoma | OR=4.08, р<0.01 (GG) | Wu J. et al., 2011 |
| 20 | rs17577 | *MMP9* | 23/23 | gastric cancer | OR=3.35, р<0.05 (haplotype CAA rs3918242-rs17576-rs17577) | Okada R. et al., 2017 |
