# Supplementary material for: Association of the functionally significant polymorphisms of the MMP9 gene with H. pylori-positive gastric ulcer in the Caucasian population of Central Russia
Source: PLoS One. 2021 Sep 7;16(9):e0257060. doi: 10.1371/journal.pone.0257060 (PMC8423286; doi:10.1371/journal.pone.0257060)
Supplement: S3 Table — (DOC) [file pone.0257060.s003.doc]

S3 table

The allele and genotype frequencies of the studied *MMP* gene SNPs in the GU patients and control group

| Chr | SNP | Gene | Minor allele | Major allele | Minor allele frequency | Number of the studied chromosomes | Genotype distribution* | H_o_ | H_e_ | Р_HWE_ |
| --- | --- | --- | --- | --- | --- | --- | --- | --- | --- | --- |
| GU patients (n=434) | | | | | | | | | | |
| 11 | rs1940475 | *MMP8* | T | C | 0.480 | 860 | 109/195/126 | 0.454 | 0.499 | 0.219 |
| 11 | rs1799750 | *MMP1* | 2G | 1G | 0.442 | 824 | 92/180/140 | 0.437 | 0.493 | 0.120 |
| 11 | rs679620 | *MMP3* | T | C | 0.494 | 856 | 102/219/107 | 0.512 | 0.500 | 0.871 |
| 16 | rs243865 | *MMP2* | T | C | 0.245 | 840 | 32/142/246 | 0.338 | 0.370 | 0.197 |
| 20 | rs3918242 | *MMP9* | T | C | 0.138 | 848 | 8/101/315 | 0.238 | 0.238 | 1.000 |
| 20 | rs3918249 | *MMP9* | C | T | 0.402 | 844 | 59/221/142 | 0.524 | 0.481 | 0.198 |
| 20 | rs17576 | *MMP9* | G | A | 0.397 | 860 | 67/209/156 | 0.484 | 0.479 | 1.000 |
| 20 | rs3787268 | *MMP9* | A | G | 0.241 | 860 | 14/179/237 | 0.416 | 0.366 | 0.060 |
| 20 | rs2250889 | *MMP9* | G | C | 0.118 | 856 | 8/85/335 | 0.199 | 0.208 | 0.501 |
| 20 | rs17577 | *MMP9* | A | G | 0.139 | 840 | 6/105/309 | 0.250 | 0.240 | 0.773 |
| control group (n=347) | | | | | | | | | | |
| 11 | rs1940475 | *MMP8* | T | C | 0.494 | 692 | 92/158/96 | 0.456 | 0.499 | 0.107 |
| 11 | rs1799750 | *MMP1* | 2G | 1G | 0.479 | 678 | 85/155/99 | 0.457 | 0.499 | 0.127 |
| 11 | rs679620 | *MMP3* | T | C | 0.504 | 690 | 89/170/86 | 0.492 | 0.500 | 0.829 |
| 16 | rs243865 | *MMP2* | T | C | 0.249 | 686 | 24/123/196 | 0.358 | 0.374 | 0.470 |
| 20 | rs3918242 | *MMP9* | T | C | 0.169 | 686 | 11/94/238 | 0.274 | 0.281 | 0.699 |
| 20 | rs3918249 | *MMP9* | C | T | 0.377 | 690 | 57/146/142 | 0.423 | 0.469 | 0.067 |
| 20 | rs17576 | *MMP9* | G | A | 0.361 | 692 | 45/159/142 | 0.456 | 0.461 | 0.907 |
| 20 | rs3787268 | *MMP9* | A | G | 0.207 | 690 | 14/115/216 | 0.333 | 0.328 | 0.870 |
| 20 | rs2250889 | *MMP9* | G | C | 0.123 | 684 | 9/66/267 | 0.193 | 0.215 | 0.072 |
| 20 | rs17577 | *MMP9* | A | G | 0.172 | 680 | 13/91/236 | 0.267 | 0.284 | 0.255 |

Supplementary table 3 (continued)

The allele and genotype frequencies of the studied *MMP* gene SNPs in the GU patients and control group

| Chr | SNP | Gene | Minor allele | Major allele | Minor allele frequency | Number of the studied chromosomes | Genotype distribution* | H_o_ | H_e_ | Р_HWE_ |
| --- | --- | --- | --- | --- | --- | --- | --- | --- | --- | --- |
| *H. pylori*-positive GU patients (n=196) | | | | | | | | | | |
| 11 | rs1940475 | *MMP8* | T | C | 0.477 | 388 | 51/83/60 | 0.428 | 0.499 | 0.222 |
| 11 | rs1799750 | *MMP1* | 2G | 1G | 0.424 | 368 | 36/84/64 | 0.457 | 0.488 | 0.527 |
| 11 | rs679620 | *MMP3* | T | C | 0.474 | 384 | 40/102/50 | 0.531 | 0.499 | 0.682 |
| 16 | rs243865 | *MMP2* | T | C | 0.232 | 380 | 14/60/116 | 0.316 | 0.356 | 0.258 |
| 20 | rs3918242 | *MMP9* | T | C | 0.161 | 380 | 2/57/131 | 0.300 | 0.270 | 0.453 |
| 20 | rs3918249 | *MMP9* | C | T | 0.444 | 376 | 29/109/50 | 0.580 | 0.494 | 0.095 |
| 20 | rs17576 | *MMP9* | G | A | 0.448 | 384 | 36/100/56 | 0.521 | 0.495 | 0.683 |
| 20 | rs3787268 | *MMP9* | A | G | 0.289 | 388 | 12/88/94 | 0.454 | 0.411 | 0.457 |
| 20 | rs2250889 | *MMP9* | G | C | 0.123 | 380 | 4/41/145 | 0.216 | 0.225 | 0.637 |
| 20 | rs17577 | *MMP9* | A | G | 0.167 | 372 | 2/58/126 | 0.312 | 0.278 | 0.452 |
| *H. pylori*-negative GU patients (n=238) | | | | | | | | | | |
| 11 | rs1940475 | *MMP8* | T | C | 0.483 | 472 | 58/112/66 | 0.475 | 0.499 | 0.584 |
| 11 | rs1799750 | *MMP1* | 2G | 1G | 0.456 | 456 | 56/96/76 | 0.421 | 0.496 | 0.130 |
| 11 | rs679620 | *MMP3* | T | C | 0.511 | 472 | 62/117/57 | 0.496 | 0.500 | 0.855 |
| 16 | rs243865 | *MMP2* | T | C | 0.256 | 460 | 18/82/130 | 0.357 | 0.381 | 0.469 |
| 20 | rs3918242 | *MMP9* | T | C | 0.120 | 468 | 6/44/184 | 0.188 | 0.211 | 0.207 |
| 20 | rs3918249 | *MMP9* | C | T | 0.367 | 468 | 30/112/92 | 0.479 | 0.465 | 0.844 |
| 20 | rs17576 | *MMP9* | G | A | 0.355 | 476 | 31/107/100 | 0.450 | 0.458 | 0.936 |
| 20 | rs3787268 | *MMP9* | A | G | 0.201 | 472 | 2/91/143 | 0.386 | 0.322 | 0.042 |
| 20 | rs2250889 | *MMP9* | G | C | 0.109 | 476 | 4/44/190 | 0.185 | 0.195 | 0.627 |
| 20 | rs17577 | *MMP9* | A | G | 0.118 | 468 | 4/47/183 | 0.201 | 0.207 | 0.646 |

Note: * minor allele homozygotes / heterozygotes / major allele homozygotes
