# Supplementary material for: Association of the functionally significant polymorphisms of the MMP9 gene with H. pylori-positive gastric ulcer in the Caucasian population of Central Russia
Source: PLoS One. 2021 Sep 7;16(9):e0257060. doi: 10.1371/journal.pone.0257060 (PMC8423286; doi:10.1371/journal.pone.0257060)
Supplement: S4 Table — (DOCX) [file pone.0257060.s004.docx]

S4 Table

Effect of the *MMP9* gene polymorphisms on affinity of the DNA regulatory motifs

(according to HaploReg, v4.1, http://archive.broadinstitute.org/mammals/haploreg/haploreg.php)

| SNPs | transcription factors | log-odds (LOD) scores | | ΔLOD | increases (↑) or decreases (↓)  affinity motifs |
| --- | --- | --- | --- | --- | --- |
|  |  | reference (ref) allele | alternative (alt) allele |  |  |
| rs3918242  (ref:C, alt:T) | Ahr:Arnt | 11.3 | -0.6 | -11.9 | ↓ |
|  | E2F | 6.2 | 10.2 | 4.0 | ↑ |
|  | HIF1 | 7.2 | -4.7 | -11.9 | ↓ |
|  | Myc | 10.4 | -0.9 | -11.3 | ↓ |
| rs3918249  (ref:T, alt:C) | Arid3a | 11.0 | 10.3 | -0.7 | ↓ |
|  | Hmx | 9.7 | 11.6 | 1.9 | ↑ |
|  | Hoxb8 | 9.5 | 12.4 | 2.9 | ↑ |
|  | Pax-5 | 12.7 | 8.8 | -3.9 | ↓ |
| rs17576  (ref:A, alt:G) | Pax-4 | 11.6 | 9.5 | -2.1 | ↓ |
| rs3787268  (ref:G, alt:A) | HDAC2 | 11.6 | 13.2 | 1.6 | ↑ |
|  | Mef2 | -5.5 | 6.5 | 12.0 | ↑ |
|  | Pou1f1 | 9.9 | 11.7 | 1.8 | ↑ |
|  | Sox | 5.9 | 14.2 | 8.3 | ↑ |
|  | Zfp105 | 9.6 | 13.1 | 3.5 | ↑ |
|  | p300 | 11.8 | 12.7 | 0.9 | ↑ |
| rs2250889  (ref:G, alt:C) | NRSF | 6.5 | 11.1 | 4.6 | ↑ |

*Note:* ΔLOD = LOD (alt) - LOD (ref). A negative value indicates the increased affinity of this motif for the reference allele, while a positive value suggests the respective increase for the alternative allele.
